# The Association between Plasma 25-Hydroxyvitamin D and Subgroups in Age-Related Macular Degeneration: A Cross-Sectional Study

**DOI:** 10.1371/journal.pone.0070948

**Published:** 2013-07-29

**Authors:** Amardeep Singh, Mads Krüger Falk, Yousif Subhi, Torben Lykke Sørensen

**Affiliations:** Department of Ophthalmology, Clinical Eye Research Unit, Copenhagen University Hospital, Roskilde, Denmark and University of Copenhagen, Copenhagen, Denmark; Saitama Medical University, Japan

## Abstract

**Objectives:**

To evaluate potential differences in plasma 25-hydroxyvitamin in subtypes of age-related macular degeneration (AMD), and in patients in Clinical Age-Related Maculopathy Staging (CARMS) group 5 with or without subretinal fibrosis.

**Methods:**

This single-center cross-sectional study included 178 participants during a period of 20 months. Ninety-five patients belonged to CARMS 5; twelve belonged to CARMS 4; twenty-two belonged to CARMS 2 or 3; and 49 individuals did not have AMD (CARMS 1). Following a structured interview, a detailed bilateral retinal examination was performed and participants were allocated to their respective subgroups in accordance with the Clinical Age-Related Maculopathy Staging system. Plasma 25-hydroxyvitamin D2 and D3 were analyzed using liquid chromatography-tandem mass spectrometry. Genomic DNA was extracted from leukocytes and genotyped for single nucleotide polymorphisms (SNPs) in the vitamin D metabolism. Differences in plasma 25-hydroxyvitamin D were determined in the subgroups as well as between patients in CARMS 5 with or without subretinal fibrosis.

**Results:**

Plasma 25-hydroxyvitamin D was comparable in patients across CARMS groups 1 to 5 (p = 0.83). In CARMS 5, the presence of subretinal fibrosis was associated with significantly lower concentrations of 25-hydroxyvitamin D as compared to the absence of subretinal fibrosis (47.2 versus 75.6 nmol/L, p<0.001). Patients in CARMS 5 with subretinal fibrosis were more likely to have insufficient levels of 25-hydroxyvitamin D compared to patients without subretinal fibrosis (p = 0.006). No association was found between the SNPs rs10877012, rs2228570, rs4588, or rs7041 and AMD subgroups or plasma 25-hydroxyvitamin levels.

**Conclusions:**

This study suggests that the presence of subretinal fibrosis in patients belonging to CARMS 5 may be associated with a poor vitamin D status. Our observations warrant further investigation into the role of vitamin D in the development of subretinal fibrosis.

## Introduction

Age-related macular degeneration (AMD) is the leading cause of visual impairment in elders over 60 years of age [1]–[4]. The early stages of the disease may be asymptomatic and are clinically characterized by drusen formation and changes in the retinal pigment epithelial (RPE) cells. In some patients, atrophy and/or choroidal neovascularization (CNV) with or without subretinal fibrosis may develop resulting in significant loss of central vision. While the vascular component of CNV often responds to treatment with intravitreal anti-vascular growth factor compounds (anti-VEGF), the fibrous tissue component of CNV mostly appears not to do so and often increases in prominence after treatment, with associated irreversible damage to the retinal architecture and severe vision loss. Interactions between genetic and environmental factors are believed to underlie the pathogenesis, but the precise mechanisms remain poorly understood [5], [6]. Oxidative stress and alterations in the immune system appear to be critical factors in the development of AMD [7], [8]. Dysregulation of the immune system favors an environment consisting of chronic inflammation, angiogenesis, and fibrosis.

Vitamin D is a circulating steroid hormone which exerts its effects by binding to the intracellular vitamin D receptor (VDR) and possesses properties that counteract inflammation, angiogenesis, oxidative stress, and fibrosis [9]–[12]. Vitamin D deficiency has been associated with several pathologies, and may also be involved in the pathogenesis of AMD. So far, findings on the relationship between vitamin D status and AMD have been inconsistent. Provided that vitamin D is an inhibitor of angiogenesis and fibrosis, it is surprising that no association has been reported between neovascular AMD and low vitamin D levels. Since substantial clinical heterogeneity is seen in neovascular AMD, potential changes in vitamin D levels may be overshadowed when failing to separate patients where subretinal fibrosis is present from patients where fibrosis is not present. Vitamin D deficiency has been linked to different conditions characterized by fibrosis, and laboratory studies have identified vitamin D as an inhibitor of fibrosis-inducing factors [10], [13]–[15]. Why only some patients with AMD develop subretinal fibrosis remains unknown. We have recently reported alterations in the complement system to be associated with subretinal fibrosis [16]. Therefore, we set out to evaluate two hypotheses; 1) whether plasma vitamin D concentrations were different in patients with subtypes of AMD, compared to individuals without AMD; and 2) whether plasma vitamin D concentrations in patients in CARMS 5 were different in those with subretinal fibrosis and those without. As certain single-nucleotide polymorphisms (SNPs) in genes related to the vitamin D metabolism have previously been reported to influence the level of optimal vitamin D concentrations required to reduce disease outcomes, we also sought to control for these SNPs in patients with CARMS 5 [17].

## Methods

### Study subjects

**Figure 1:**
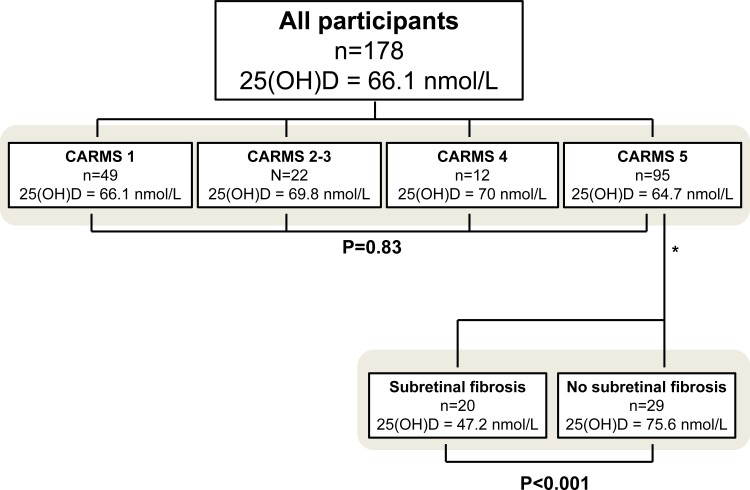
Number of participants and mean plasma 25-hydroxyvitamin D, 25(OH)D in subgroups of age-related macular degeneration. Participants were categorized into subgroups after a thorough retinal examination. Please note that when comparing vitamin D status in patients in CARMS 5 with or without subretinal fibrosis, 46 previously treated patients with neovascular AMD were excluded from analysis (denoted by asterix).

During the course of inclusion (n = 20 months) in this single-center study, we asked patients who had been referred to our department because of AMD, or patients with previously confirmed diagnosis of AMD to participate. The referred patients were subsequently subjected to a thorough retinal examination to verify the presence of AMD. The maculae of all participants were graded according to the Clinical Age-Related Maculopathy Staging (CARMS) system [18]. Thus, participants were divided into the following groups: CARMS 1 (no drusen or <10 small drusen without pigment abnormalities); CARMS 2 and 3 (approximately ≥10 small drusen or <15 intermediate drusen, pigment abnormalities associated with age-related maculopathy, or approximately ≥15 intermediate drusen or any large drusen with or without retinal pigment epithelial detachment), CARMS 4 (geographic atrophy with involvement of the macular center, or noncentral geographic atrophy at least 350 µm in size; CARMS 5 (exudative AMD, including nondrusenoid pigment epithelial detachments, serous or hemorrhagic retinal detachments, choroidal neovascular membrane with subretinal or sub-RPE hemorrhages or fibrosis, or scars consistent with treatment of AMD). In order to investigate the association between subretinal fibrosis and vitamin D concentration, we subdivided patients in CARMS 5 into those who had subretinal fibrosis and those who did not, and compared vitamin D concentrations in these two groups. To minimize the effects of disease duration on the development of subretinal fibrosis when comparing the vitamin D concentrations between patients in CARMS 5 with or without subretinal fibrosis, we only included patients who were newly referred to our clinic, as opposed to patients who were already undergoing treatment for choroidal neovascularization at the time of inclusion (Figure 1). The exclusion criteria were: immune modulating treatment; chronic kidney, liver or parathyroid disease; cancer; inflammatory or autoimmune diseases; intravitreal anti-VEGF therapy within the last 30 days; non-gradable maculae, or age below 60 years. All, but two participants were of Caucasian origin.

### Ethics statement

The study has been approved by the Regional Committee of Ethics in Research of the Region of Zealand (SJ-142) and verbal and written informed consent was obtained from all participants prior to inclusion. The described research adhered to the tenets of the Declaration of Helsinki.

### Clinical data

All participants were subjected to a structured interview concerning previously diagnosed and current medical conditions, medication status (including over-the-counter drugs, supplements and natural remedies), and lifestyle. Body weight and height were noted or measured and used to calculate the body mass index. Depending on their smoking habits, participants were grouped as current smokers, former smokers (more than 100 cigarettes during their life), or never smokers. Those who reported quitting within the last year were grouped as current smokers [19]. Harmful alcohol consumption was defined according to updated guidelines from the Danish National Board of Health. Thus, alcohol consumption was labeled as being above the recommended level if the usage was higher than 7 units of alcohol a week for women and 14 units a week for men [20]. The participants were categorized as being either physical active or inactive based on a single-sentence questionnaire as proposed by Schectman and co-workers (translated into Danish by the authors): *Do you currently participate in any regular activity or program (either on your own or in a formal class) designed to improve or maintain your physical fitness* [21]?

### Retinal imaging and grading

The retinal diagnosis was made by trained ophthalmologists using bilateral ophthalmoscopic fundus examination, digital color fundus photography (Carl Zeiss, Germany), Spectral-Domain Optical Coherence Tomography and autofluorescence imaging (Spectralis HRA-OCT, SLO Heidelberg Engineering, Heidelberg, Germany). Fluorescein and indocyanine green angiography was performed following blood sampling to avoid interference in patients suspected of having neovascular AMD [22]. Two ophthalmologists graded the maculae independently of each other blinded from any available data on 25-hydroxyvitamin D.

### Subretinal fibrosis

Subretinal fibrosis refers to the reparative changes in response to retinal tissue injury leading to formation of excess fibrous connective tissue formation below the retina. The presence of subretinal fibrosis in the macula justifies classification into CARMS 5 and is thus a feature of late AMD. The diagnosis of subretinal fibrosis was made if it was present at the initial visit in any form or size and in any distance to the fovea in either fundus examination or digital photography (white, fibrous tissue under the retina), or SD-OCT (dense hyperreflective area between the RPE and neurosensory area or beneath the RPE). Subretinal fibrin exudation was not included in the definition.

### Plasma vitamin D measurement

One venous blood sample (4 ml) was obtained in an evacuated gel tube for each participant. Within two hours of sampling, the serum was isolated by centrifugation and stored for at maximum of 6 months at −80°C. Previous studies report that the stability of 25-hydroxyvitamin D is unaffected by storage time [23]. The samples were transported to National University Hospital Rigshospitalet where both plasma 25-hydroxyvitamin D2 and D3 concentrations were measured using liquid chromatography-tandem mass spectrometry (Waters UPLC-TQD LC-MS/MS, Milford Massachusetts, USA). The maximum coefficient of variation was 10–12% for 25-hydroxyvitamin D2 and 8–10% for 25-hydroxyvitamin D3.

Vitamin D status was considered insufficient when 25-hydroxyvitamin D was equal to or below 50 nmol/L, and sufficient when 25-hydroxyvitamin D was above 50 nmol/L [24]. To convert to nmol/L to ng/mL, divide by 2.496.

### DNA extraction and genotyping

Venous blood for genotype analysis was obtained in a 3 ml tube containing ethylenediamine-tetraacetic acid coagulant and posted to the Kennedy Center (Glostrup, Denmark) where genomic DNA was extracted from leukocytes using Chemagic Magnetic Separation Module I (Chemagen, Baesweiler, Germany). Samples were then sent for genotyping to LGC Genomics Ltd (Herts, United Kingdom) where genotyping was performed using in-house KASP™ (Kompetetive Allele Specific Polymerase chain reaction) genotyping SNP-line system which subsequently resulted in KASP assays.

The following SNPs were analyzed: rs10877012 (25-hydroxyvitamin D3 1-alpha-hydroxylase, CYP27B1, which when defective may reduce the efficiency of hydroxylation of 25-hydroxyvitamin D to the active 1,25-dihydroxyvitamin D resulting in a scenario where plasma 25-hydroxyvitamin D concentrations appear normal despite subnormal concentrations of 1,25-dihydroxyvitamin D), rs2228570 (vitamin D receptor, which when defective may alter the pattern of vitamin D-mediated gene activation affecting enzymes involved in production and elimination of 25-hydroxyvitamin D), and rs4588 and rs7041(vitamin D binding proteins, which when defective may alter the ratio of the free fraction of 25-hydroxyvitamin D and VDBP-bound fraction leading to diminished access of vitamin D to target sites despite normal plasma 25-hydroxyvitamin D concentrations). The SNPs were tested for deviation from the Hardy-Weinberg Equilibrium.

### Statistical analysis

Statistical analysis was performed using SPSS 20 for Windows (IBM, Chicago, IL, USA). When comparing demographics and clinical data across CARMS group 1–5, One-way Analysis of Variance (ANOVA) was used for continuous variables with a normal distribution (25-hydroxyvitamin D and BMI) and the average was provided as a mean with standard deviation. The Kruskal-Wallis test was applied to continuous variable that were not normally distributed (age) and the average was given as the median and the interquartile range. Categorical variables, such as gender, vitamin D supplementation, physical activity, smoking habits, alcohol consumption, and CNV type were compared using Pearson's Chi-square test or Fischer's exact test where appropriate. Multiple logistic regression analysis was used to evaluate the effect of confounders on the presence or absence of subretinal fibrosis. When comparing demographics and clinical data between patients in CARMS 5 with or without subretinal fibrosis, Student's independent t-test was used for variables with a normal distribution (25-hydroxyvitamin D concentrations and BMI), while the Mann-Whitney test was used to compare age. Categorical variables were compared using Pearson's Chi-square test. Similarly, the distribution of fibrosis versus no-fibrosis in patients with vitamin D sufficiency (nmol/L or >50 nmol/L) or insufficiency (≤50 nmol/L) was compared using Pearson's Chi-square test. The distribution of alleles across CARMS 1–5 and between fibrosis/no-fibrosis groups was evaluated using Pearson's Chi-square test. The association between SNPs and 25-hydroxyvitamin D concentrations was evaluated using ANOVA. A Chi-squre test was used to test the SNPs for deviation from the Hardy-Weinberg Equilibrium. A p-value<0.05 was considered statistically significant.

## Results

Table 1 shows the sociodemographic, lifestyle, and clinical characteristics of all included participants. There were no significant differences in the gender, BMI, vitamin D supplementation, physical activity, smoking habits or percentage of persons consuming more alcohol than recommended across the CARMS groups.

**Table 1:** Sociodemographic, lifestyle and clinical characteristics of participants (n = 178).

	CARMS 1 (n = 49)	CARMS 2/3 (n = 22)	CARMS 4 (n = 12)	CARMS 5 (n = 95)	P-value
p-25(OH)D, nmol/L (mean, SD)	66.1, 25.1	69.8, 26.6	70, 17.4	64.7, 29.5	0.83
Age, median (IQR), y	73 (68–79)	76 (69.5–83)	74 (72.5–78)	76 (70–81)	0.26
Sex					
Males (%)	53	13	5	38	
Females (%)	47	9	7	57	0.27
Body mass index (mean, SD)	27.1, 5.3	26.2, 4.1	27.6, 3.9	25.9, 4.6	0.39
Vitamin D supplement user (%yes)	26	18	42	21	0.40
Physical activity, (%yes)	58	59	75	57	0.13
Smoking habits					
Current smokers, (%yes)	8	9	17	25	0.27
Former smokers (%yes)	47	50	42	38	
Never smokers (%yes)	45	41	42	38	
Alcohol intake					
Above recommended (%yes)	16	18	25	13	0.90

Abbreviations used: SD = standard deviation, IQR = interquartile range.

Data are expressed as mean and standard deviation for continous variables (p-25-hydroxyvitamin D and body mass index) and as median and interquartile range for categorical variables (age, supplement use, physical activity, smoking habits and alcohol intake). There was a significant difference in age across groups (p<0.001). Body mass index data missing for 5 patients in CARMS 5.

**Figure 2:**
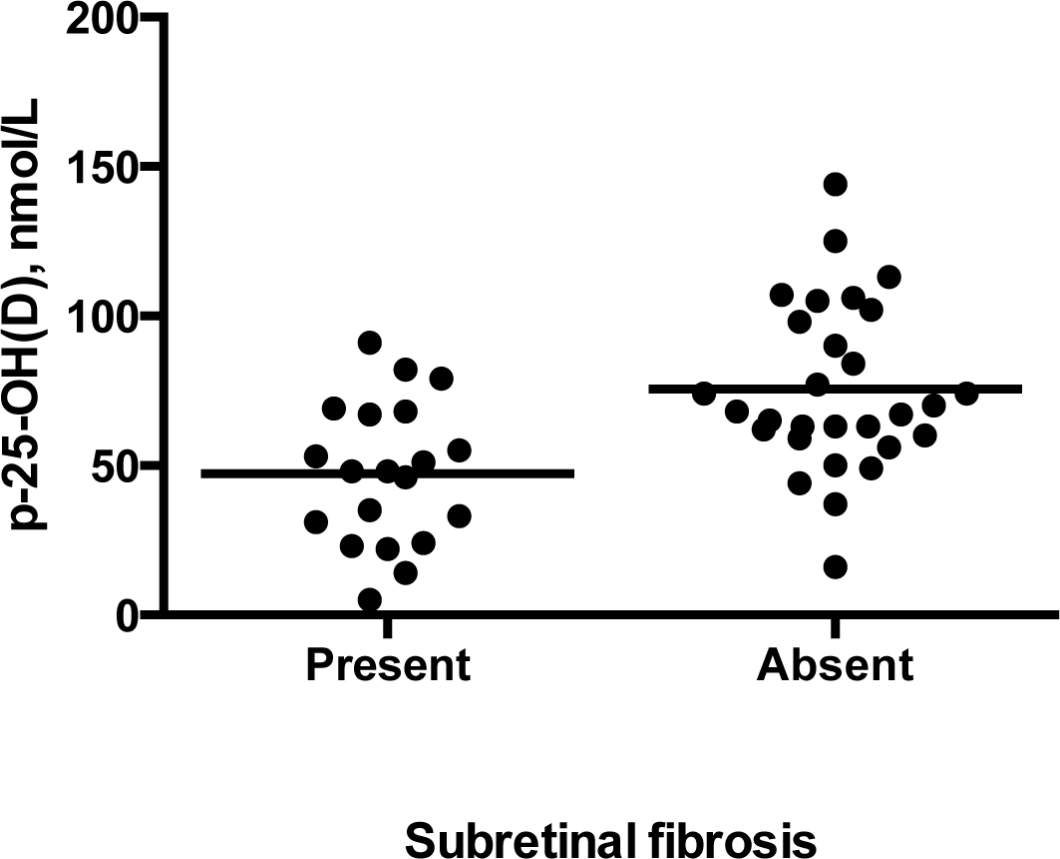
25-hydroxyvitamin D in patients in CARMS 5 with or without subretinal fibrosis. Patients with subretinal fibrosis had significantly lower plasma 25-hydroxyvitamin D concentrations compared to patients without subretinal fibrosis (p<0.001). Horizontal lines represent means.

**Figure 3:**
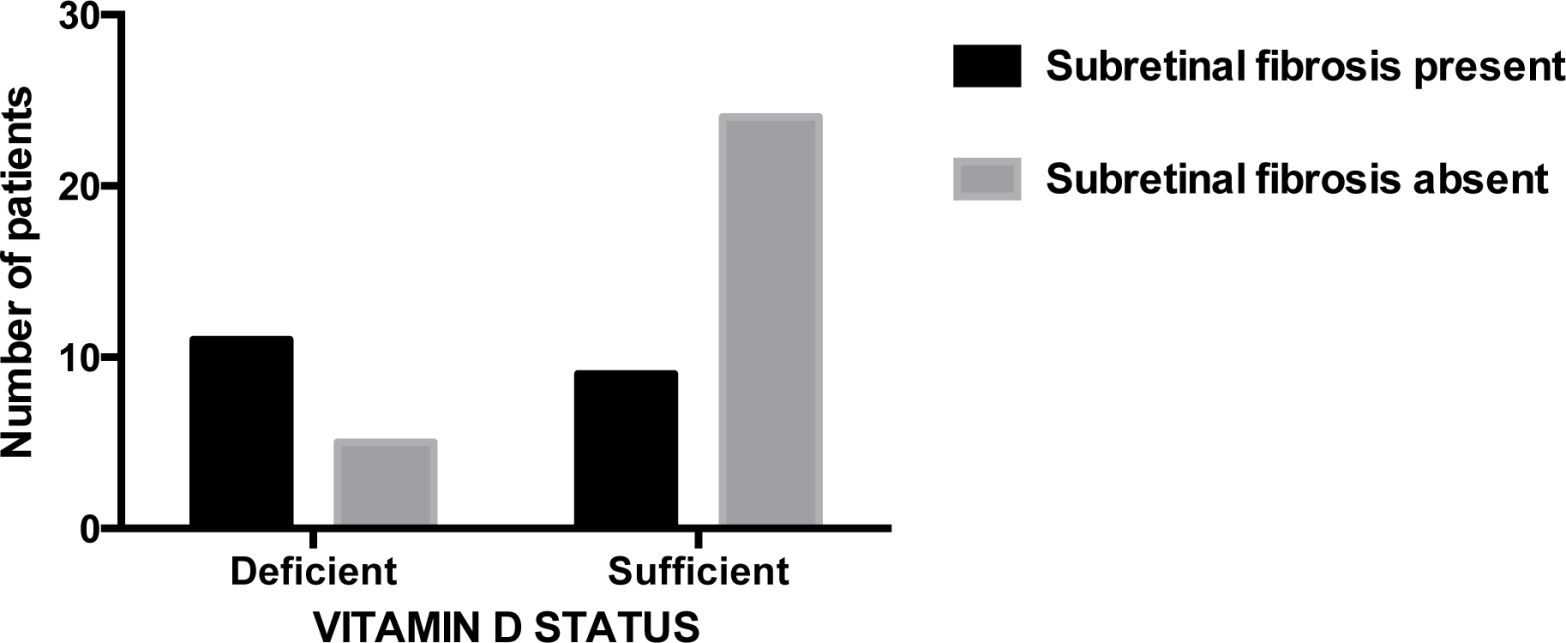
Subretinal fibrosis status in patients with vitamin D insufficiency or sufficiency. Patients without subretinal fibrosis (light filling) were more likely to be vitamin D sufficient compared to those with subretinal fibrosis (dark filling) who were more likely to be deficient (p = 0.006, Pearson's Chi-square test). Vitamin D insufficiency was defined as 25-hydroxyvitamin D≤50 nmol/L and sufficiency was defined as >50 nmol/L.

The mean 25-hydroxyvitamin D for all 178 participants was 66.1 nmol/L (standard deviation = 27.2). Comparison of 25-hydroxyvitamin D across CARMS 1 to 5 revealed no significant differences (p = 0.83, ANOVA). When patients in CARMS 5 were subdivided depending on whether or not subretinal fibrosis was observed, there was a significant difference in 25-hydroxyvitamin D concentrations (75.6 nmol/L if subretinal fibrosis was absent vs. 47.2 nmol/L if subretinal fibrosis was present (p<0.001, Student's independent t-test) (Figure 2)). As shown in figure 3, patients in CARMS 5 without subretinal fibrosis were more likely to have 25-hydroxyvitamin D concentrations above 50 nmol/L compared to patients with subretinal fibrosis, who were more likely to have vitamin D deficiency (p = 0.006, Chi-square test). Apart from a lower visual acuity in patients with subretinal fibrosis, no significant differences in patient characteristics were observed in patients with or without subretinal fibrosis (Table 2). None of the participants belonging to CARMS 1–4 had signs of subretinal fibrosis. There was no seasonal pattern of patients presenting with subretinal fibrosis (Table 3).We adjusted for potential confounders on the relation between subretinal fibrosis status and vitamin D status using a multiple logistic regression analysis with the following co-variates: (p-25-hydroxyvitamin D, age, season, smoking, alcohol consumption, CNV type, gender, physical exercise, vitamin D supplementation, body mass index, and the four mentioned SNPs). Still, vitamin D status was significantly associated with subretinal fibrosis (p = 0.01), while the co-variates were insignificant.

**Table 2:** Sociodemographics, lifestyle and clinical chacteristics for treatment-naïve patients in CARMS 5 with or without fibrosis.

	*Subretinal fibrosis*		P-value	P-value, multiple logistic regression analysis
	Present (n = 20)	Absent (n = 29)		
p-25(OH)D, nmol/L (mean, SD)	47.2 (23.9)	75.6 (28.0)	<0.001	0.011
Age, y (median, IQR)	76 (70–81.5)	78.5 (72.5–84.5)	0.23	0.53
Sex			0.69	0.52
Males, n	8	10		
Females, n	12	19		
Body mass index (mean, SD)	25.6 (5.2)	25.6 (4.4)	0.98	0.36
Supplement users (%yes)	17	31	0.27	0.39
Physical activity (%yes)	47	66	0.27	0.42
Smoking habits			0.90	0.98
Current smokers (%yes)	26	24		
Former smokers (%yes)	32	38		
Never smokers (%yes)	42	38		
Alcohol intake			0.48	0.24
Above recommended (%yes)	10	10		
Type of choroidal neovascularization*			0.46	n.a
Type 1	6	8		.
Type 2	7	13		
Best corrected visual acuity, EDTRS, median (IQR)				
Better eye	67 (57–84)	81 (76–85)	0.008	n.a
Worse eye	21 (7.75–52)	70 (55.5–76)	<0.001	n.a.

Abbreviations used: SD = standard deviation, IQR = interquartile range.

Data are expressed as mean and standard deviation for continous variables (p-25-hydroxyvitamin D and body mass index) and as median and interquartile range for categorical variables (age, supplement use, physical activity, smoking habits and alcohol intake). There was a significant difference in p-25-hydroxyvitamin D between patients with fibrosis and patients without fibrosis (p<0.001). Note that patients with neovascular AMD who were receiving anti-vascular endothelial growth factors in the past or present were excluded from this analysis. Body mass index data missing for 2 patients with subretinal fibrosis and 1 patient without subretinal fibrosis.

* Only patients with active CNV type 1 or 2 on fluorescein angiography (data not analyzed using multiple logistic regression since only 34 patients had active classic/occult CNV). The type of choroidal neovascularization was not included in the multiple logistic regression analysis as not all patients had active CNV, and best corrected visual acuity was not included because it is not the cause of subretinal fibrosis, but a result of it.

**Table 3:** Number of patients with or without subretinal fibrosis according to season.

	Winter season (October 1st–March 31st)	Summer season (April 1st–September 30th)	P-value
Fibrosis present, No. (%)	8 (16)	17 (35)	
Fibrosis absent, No. (%)	11 (22)	13 (27)	0.93

Data are expressed as number of patients and percentages of total number of patients (n = 49). There were no significant seasonal differences in the inclusion of patients with or without subretinal fibrosis. Test used: Pearson's Chi-square test. Latitude of Copenhagen University Hospital Roskilde: 55.636208.

A CNV membrane may be located between the RPE and Bruch's membrane (occult CNV, type 1), or between the RPE and retina (classic CNV, type 2) [25]. There is often some overlap between the two types of CNV. The development of subfoveal fibrosis in neovascular AMD may be associated with predominantly classic CNV [26]. In our study, 14 patients (29%) in CARMS 5 had type 1 CNV; 20 patients (41%) had type 2 CNV; eleven patients (22%) had no signs of active CNV; one patient (2%) had mixed CNV and one patient had type 1 CNV in one eye and type 2 CNV in the other; fluorescein angiography was not performed in 1 patient who had other signs of late AMD, e.g. central large disciform scar; in 1 patient signs of late AMD were detected for the first time during inclusion and the patient was subsequently referred to another ophthalmology department in Denmark for further evaluation. We found no association between CNV type and the presence of subretinal fibrosis (p = 0.46, Fischer's exact test).

No intergroup differences in allele frequency was observed across CARMS groups 1–5 or in patients in the CARMS 5 group with or without subretinal fibrosis. Moreover, 25-hydroxyvitamin D concentrations were not affected by genotype status (Table 4). None of the four SNPs deviated from Hardy-Weinberg equilibrium (rs10877012, χ^2^ = 2.93, p = 0.087; rs2228570, χ^2^ = 0.11, p = 0.74; rs4588, χ^2^ = 0.0021, p = 0.96; rs7041, χ^2^ = 3.19, p = 0.074).

**Table 4:** Genotype frequencies of participants.

	rs10877012*			rs2228570			rs4588**			rs7041***		
CARMS	GG	GT	TT	CC	CT	TT	CC	AC	AA	GG	GT	TT
1	51	33	16	37	51	12	49	43	8	27	53	20
2–3	45	41	14	50	41	9	62	33	5	27	64	9
4	58	33	8	25	58	17	42	50	8	17	75	8
5	45	42	13	33	45	22	52	40	8	24	54	21
p-value			0.93			0.48			0.95			0.71
5 without subretinal fibrosis	48	38	14	31	48	21	41	45	14	24	48	28
5 with subretinal fibrosis	47	47	5	35	30	35	50	45	5	11	63	26
p-value			0.59			0.38			0.58			0.45
p-25-hydroxyvitamin D, nmol/L (mean, SD)	65.7, 29.0	61.3, 30.8	66.0, 37.5	62.8, 38.1	63.8, 28.8	65.8, 19.8	66.3, 28.1	59.5, 25.7	73.4, 52.3	68.4, 32.3	57.6, 25.0	73.3, 36.5
P-value			0.88			0.97			0.58			0.27

Genotype frequencies given as percentages.

Abbreviations used: SD = standard deviation, L = litre.

Tests used: Chi-square test for comparison of genotype frequencies between CARMS 1–5 and between CARMS 5 with subretinal fibrosis and CARMS 5 without subretinal fibrosis, and one-way ANOVA test for comparison of plasma 25-hydroxyvitamin D concentrations between different genotypes. No significant differences were found in the genotype frequencies between CARMS 1–5, or in the 25-hydroxyvitamin D concentrations between different genotypes.

* Genotype missing for 4 patients with CARMS 5.

** Genotype missing for 1 patient with CARMS 4.

*** Genotype missing for 1 patient with CARMS 5.

## Discussion

We report an inverse association between plasma 25-hydroxyvitamin D and the presence of subretinal fibrosis in patients presenting with CARMS 5. We did not observe any differences in 25-hydroxyvitamin D concentrations between patients in CARMS 1–5, suggesting that vitamin D deficiency is specifically associated with subretinal fibrosis in AMD.

A few studies have evaluated the relationship between AMD and vitamin D status. In 2007, Parekh and co-workers were the first to demonstrate an association between early, but not late, AMD and vitamin D deficiency in a cross-sectional study [27]. This study was, however, limited by a small number of patients in the advanced AMD group (n = 10). In another study by Millen and co-workers, high serum 25-hydroxyvitamin D was found to be protective against early AMD in women younger than 75 years of age [28]. A recent retrospective study by Graffe and co-workers demonstrated that hypovitaminosis D (defined as 25-hydroxyvitamin D<50 nmol/L) was more frequent in patients with AMD, in particular the late stages, compared to controls. Participants with hypovitaminosis were more likely to have late stage AMD than those with normal vitamin D status [29]. Morrison and co-workers examined a family-based cohort consisting of 481 sibling pairs and found higher concentrations of 25-hydroxyvitamin D in unaffected individuals compared to their affected siblings but this finding did not reach statistical significance. In contrast, Golan and co-workers were unable to find any association between vitamin D concentrations and AMD in a large cross-sectional study [30]. They did not, however assign patients with AMD into clinical subgroups as detailed clinical data was unavailable. Moreover, the authors did not have access to information on vitamin D supplementation in the participants. None of the above studies differentiated between fibrotic and non-fibrotic neovascular AMD. Evidence from animal studies suggests that vitamin D may have a role in the pathogenesis of AMD. For instance, treatment with vitamin D in aged mice reduced retinal inflammation and amyloid-beta deposition which is considered to be a hallmark of aging. In addition, a reduction in the number of retinal macrophages and shifts towards an anti-inflammatory phenotype was observed [31]. Thus, the evidence linking vitamin D status with AMD is inconsistent and in need of further assessment.

Many pathways and processes involved in other diseases, such as Alzheimer's disease, cancer and atherosclerosis also appear to be involved in AMD. There is sufficient data from epidemiological, animal and laboratory studies to suggest that vitamin D deficiency is associated with a number of diseases. For example, a recent meta-analysis found that patients with Alzheimer's disease had lower serum vitamin D compared to matched controls [32]. In fact, supplementation with vitamin D improved cognition in patients with Alzheimer's disease [33]. Another recent study reported an association between Alzheimer's disease and a promoter SNP rs11568820 in the VDR [34]. In cardiovascular disease, a strong association between vitamin D and arterial disease has been reported in several studies [35], [36]. In cancer, vitamin D is currently being evaluated as a potential anti-cancer drug [37]. Vitamin D is believed to exert its protective effects by modulating the immune system, inhibiting inflammation and inhibiting angiogenesis [38]. Vitamin D may enhance T regulatory cell activity and downregulate T helper cells, T cytotoxic cells and natural killer cells [39], [40]. Animal and *in vitro* studies demonstrate that vitamin D inhibits angiogenesis by reducing the expression of VEGF, reducing the proliferation of endothelial cells and increasing the expression of platelet-derived growth factor. Moreover, vitamin D also inhibits the matrix-metallopeptidase 9, an extracellular degrading matrix protein that is suspected to play a role in choroidal neovascularization [41].

In addition to the effects described above, vitamin D is also a potent inhibitor of fibrosis. Damage to epithelial cells results in release of growth factors, such as transforming growth factor β (TGF-β), a potent promoter of fibrogenesis through modulation of fibroblast phenotype and function, myofibroblast transdifferentiation and matrix preservation [42]. Also released during damage are chemokines which recruit leukocytes that are capable of secreting TGF-β [43]. Macrophage-derived TGF-β1 is thought to promote fibrosis by directly activating resident mesenchymal cells and epithelial cells, which then differentiate into collagen-producing myofibroblasts [44]. TGF-β is upregulated and activated in numerous fibrotic diseases. A study found that TGF-β1, along with platelet count, may be an early indicator of bone marrow fibrosis [45]. Interestingly, the study also reported an inverse relationship between TGF-β1 and vitamin D concentrations [45]. In lung tissue, vitamin D inhibits TGF-β1 stimulated pro-fibrotic changes in lung fibroblasts and epithelial cells [15]. Halder and co-workers were able to suppress TGF-β3 induced fibrosis-related protein expression in immortalized human uterine leiomyoma cells with vitamin D3 [13]. Experimental studies have recognized vitamin D as an inhibitor of myocardial fibrosis [10], [46]. In the liver, low serum concentrations of vitamin D are related to severe fibrosis and low responsiveness to therapy [14]. Lower concentrations of vitamin D were also found in systemic sclerosis and skin fibrosis [47]. When active vitamin D was added to mesenchymal multipotent cells primed with 5′-azacytidine to induce a fibrotic phenotype, a decrease in expression of TGF-β1 and plasminogen activator inhibitor (PAI, another fibrotic factor) was observed [48]. When mouse embryonic fibroblasts were exposed to proinflammatory factors, such as tumor necrosis factor-α and lipopolysaccharide, a marked induction of PAI was inhibited by vitamin D, suggesting a role of both TGF-β and PAI-1 in inhibition of fibrosis [49]. Taken together, there is ample evidence to suggest that vitamin D plays a role in the pathogenesis of several fibrotic diseases. Our findings of reduced vitamin D concentrations in patients with subretinal fibrosis are thus in line with previous findings.

It is important to consider factors like dietary supplementation and genotype status when comparing vitamin D concentrations in disease states, as both may influence vitamin D status. In the Beaver Dam study, Klein and co-workers found no association between dietary intake of vitamin D and advanced AMD [50]. In contrast, Seddon and co-workers reported higher dietary intakes of vitamin D in the twins with early AMD compared to co-twins with more severe AMD, adjusted for smoking and age [51]. The study was specifically designed to focus on the dietary intake of vitamin D, and therefore did not complement the findings with concentrations of circulating vitamin D. In our study, patients taking vitamin D supplements had similar concentrations of 25-hydroxyvitamin D as patients not taking supplements and no significant differences were observed in supplement use across the various subgroups of AMD, suggesting that it was the level of 25-hydroxyvitamin D rather than use of supplementation that was associated with subretinal fibrosis. Anyhow, data on dietary supplementation should be interpreted with caution as supplementation with vitamin D impacts 25-hydroxyvitamin D concentrations in a non-linear fashion with considerable inter-individual variation [52]. In a systematic review, McGrath and co-workers identified four SNPs which may influence vitamin D concentrations: 1) one SNP in CYP27B1 (rs10877012) which codes for the 1-α-hydroxylase, 2) one SNP in the VDR (rs2228570), and 3) two SNPs in the vitamin D binding proteins (group-specific components) (rs4588 and rs7041) [17]. In light of these findings, it is possible that the required level of 25-hydroxyvitamin D may depend on the specific genotype. In fact, single point variants in CYP24A1 (1,25-dihydroxyvitamin D3 24-hydroxylase, a catabolic enzyme in the vitamin D pathway) have been found to influence AMD risk [53]. We controlled for the above mentioned SNPs in our study and found no significant association between allele frequency and the different clinical groups of AMD or between patients in CARMS 5 with or without fibrosis. These four SNPs did not affect vitamin D concentrations, suggesting that our findings linking subretinal fibrosis with low vitamin D concentrations are independent of genotypes.

Some limitations of this study need to be considered. Firstly, we cannot infer anything about the cause or effect relationship between vitamin D concentrations and subretinal fibrosis in CARMS 5 as our findings are based on cross-sectional observations. We did not estimate the amount of vitamin D that patients were obtaining from food or sunlight exposure, the lack or excess of which could have affected our results. Although, it is unclear how much factors like food fortification, sunlight exposure or latitude would have affected our results, as the purpose of this study was to identify relative, and not absolute intergroup differences. Also, there is a possibility of recall bias and compliance with regards to vitamin D intake and physical activity. All, but two subjects were of Caucasian origin which limits the possibility to generalize to other ethnic groups. Physical activity was determined using a single question which categorizes participants into physically active and physically inactive, but does not quantify the amount of activity. We did not distinguish between outdoor and indoor activity, the ratio of which could also influence vitamin D concentrations. Since vitamin D concentrations may fluctuate in an individual over time, it could be problematic that we measured vitamin D status from a single measurement of 25-hydroxyvitamin D. However, it has previously been documented that there is only a moderate intraindividual variation in 25-hydroxyvitamin D in postmenopausal women over 5 years; thus the use of a one-time measure of 25-hydroxyvitamin D may be justified in studies with a follow-up of 5 or less years [54]. Finally, we did not have data regarding the duration of disease prior to diagnosis and thus, we cannot rule out the possibility that patients with subretinal fibrosis had been affected with AMD for a longer period than patients without. Patients referred to our department with suspected neovascular AMD are typically examined within 14 days.

In conclusion, this observational study suggests an inverse association between low 25-hydroxyvitamin D and subretinal fibrosis in patients presenting with CARMS 5. Our findings may help understand why previous studies on the association between vitamin D concentrations and AMD have reported conflicting results and also stresses the importance of thorough clinical grading of patients with this extremely heterogenic disease when looking for differences that may help understand underlying mechanisms. Our observations warrant further investigation into the role of vitamin D status and supplementation in the development of subretinal fibrosis in late AMD.
